# RETRACTION: LncRNA TUG1 Relieves Renal Mesangial Cell Injury by Modulating the miR‐153‐3p/Bcl‐2 Axis in Lupus Nephritis

**DOI:** 10.1002/iid3.70193

**Published:** 2025-04-11

## Abstract

**RETRACTION:** L. Liu, Y. Zhang and L. Zhong, “LncRNA TUG1 Relieves Renal Mesangial Cell Injury by Modulating the miR‐153‐3p/Bcl‐2 Axis in Lupus Nephritis,” *Immunity, Inflammation and Disease* 11, no. 4 (2023): e811, https://doi.org/10.1002/iid3.811.

The above article, published online on 12 April 2023 in Wiley Online Library (wileyonlinelibrary.com), has been retracted by agreement between the journal Editor‐in‐Chief, Marc Veldhoen; and John Wiley & Sons Ltd. The retraction has been agreed upon following an investigation into concerns raised by a third party. The investigation revealed that the LPS+miR‐153‐3p inhibitor flow cytometry plot presented in Figure 6B has been found duplicated in another article by a different author group representing different scientific conditions. The authors provided an explanation and some data but this was not deemed sufficient. The editors have lost confidence in the data presented and consider the conclusions to be compromised. The authors disagree with the retraction.

**RETRACTION:** L. Liu, Y. Zhang and L. Zhong, “LncRNA TUG1 Relieves Renal Mesangial Cell Injury by Modulating the miR‐153‐3p/Bcl‐2 Axis in Lupus Nephritis,” *Immunity, Inflammation and Disease* 11, no. 4 (2023): e811, https://doi.org/10.1002/iid3.811.

The above article, published online on 12 April 2023 in Wiley Online Library (wileyonlinelibrary.com), has been retracted by agreement between the journal Editor‐in‐Chief, Marc Veldhoen; and John Wiley & Sons Ltd. The retraction has been agreed upon following an investigation into concerns raised by a third party. The investigation revealed that the LPS+miR‐153‐3p inhibitor flow cytometry plot presented in Figure 6B has been found duplicated in another article by a different author group representing different scientific conditions. The authors provided an explanation and some data but this was not deemed sufficient. The editors have lost confidence in the data presented and consider the conclusions to be compromised. The authors disagree with the retraction.

